# To Mars through LEO: How commercial space travel will change exploration‐enabling research

**DOI:** 10.1113/EP092422

**Published:** 2025-05-19

**Authors:** Christopher Puhl, Michail Magkos

**Affiliations:** ^1^ Directorate of Human and Robotic Exploration, European Space Agency Space Applications Services NV/SA for European Space Agency Noordwijk Netherlands; ^2^ Division of Ergonomics, Department of Biomedical Technology and Health Systems, School of Engineering Sciences in Chemistry, Biotechnology, and Health Royal Institute of Technology KTH Stockholm Sweden

## 1 INTRODUCTION

The Crew dragon capsule stacked atop the Falcon 9 disappeared above the clouds carrying the Axiom‐3 crew (Box 1). It was mid‐January 2024, and the mission that would carry the first European project astronaut aboard a commercial spaceflight had just commenced operations. Although the previous 8 months had been a flurry of activity preparing the mission on a swift and compressed timeline, the sonic boom that echoed across Merritt Island seemed to be an audible demarcation of a new time. Commercial human spaceflight on a larger scale had arrived; the pace was beginning to increase, and space was nearer than ever. The future? More flights, more platforms, more options and more throughput. The European Space Agency had taken the first steps into this new and evolving Low Earth Orbit (LEO) landscape. National agencies, industry and the science community seek to use these new opportunities as a springboard with eyes cast further into the distance: Moon, Mars, and Deep Space.

**Box 1. Axiom‐3: A commercial mission overview**

The Muninn mission, launched as part of the European Space Agency's (ESA) participation in the Axiom‐3 flight, represents a landmark effort toward leveraging commercial pathways for European human spaceflight. Marking the first‐ever commercial human spaceflight mission sponsored by ESA, Muninn featured astronaut Marcus Wandt, the first Project Astronaut to emerge from ESA's 2022 Astronaut Reserve, and the second Swedish national in space. Highlighting both agility and pragmatism, the mission transitioned from announcement to launch in only 7 months, making Marcus Wandt the fastest‐trained astronaut in history. The mission's compressed timeline required parallelization and increased flexibility in planning and execution, demonstrating ESA's ability to effectively manage complex operations alongside national partners like the Swedish National Space Agency (SNSA) and private entities such as Axiom Space. ESA's strategic role extended beyond traditional frameworks, acting not only as validator but also as a proactive mission operator – balancing international standards with national scientific priorities. During his 18 days aboard the ISS, Marcus conducted over 20 experiments, dedicating more than 100 h to microgravity research and technological demonstrations. ESA Director General Josef Aschbacher emphasized the significance of the Muninn mission, noting it as a decisive step toward diversifying Europe's access to space and gaining essential experience in a rapidly evolving, commercially oriented human spaceflight landscape. By successfully implementing novel concepts like Project Astronauts and tapping into the potential of commercially brokered flights, ESA and its member states have positioned themselves to capitalize on emerging opportunities in the post‐ISS era, potentially reshaping Europe's approach to space exploration.

Key concepts of the Muninn mission are the following.

Astronaut reserve: ESA's concept allowing member states to expand their astronaut pool beyond standard selections, providing trained astronauts without assigned flights who can participate via commercial partnerships.

Project astronaut: ESA's program utilizing astronauts from the Reserve, enabling rapid, flexible assignment to missions brokered through commercial entities.

Orbital Architecture activity: The authors were involved in this research activity, utilising a structured, modular experiment conducted during the mission, comprising feasibility assessments, detailed requirement definitions, subsystem development, verification, and certification, designed to minimize risks and ensure robust scientific outcomes in a fast‐paced commercial mission environment.

## 2 TOWARDS A COMMERCIAL LOW EARTH ORBIT POST‐ISS

The International Space Station (ISS) currently sits as the locus for space‐based life sciences research in LEO, only recently being joined by the Tiangong Space Station. The ISS has been continuously manned for more than two decades, and there have been over 4000 studies carried out on board it by research groups from all over the world. Here we have observed many of the deleterious effects of spaceflight on the human body: fluid shifts, headaches, disorientation, cognitive disturbances, muscle and bone loss, space‐associated neurocular syndrome (SANS), as well as a host of other effects (Cowings et al., 2018; Demontis et al., 2017; Kesserwani, 2021; Yin et al., 2023). These problems arise as a response to the combined stressors of spaceflight – microgravity, radiation, isolation and confinement. Although much work has been done, there remains a lack of precise understanding of the pathways and molecules mediating these effects, not to mention effective countermeasures to keep crew safe during the round trip to Mars (Box 2). Continued study, increasing the number of subjects, and testing countermeasures remain critical objectives in LEO before taking the trip to deep space, and continued access to crewed platforms in LEO are a necessary component.

**Box 2. LEO: Our launchpad to Mars**

Traveling to Mars presents a series of physical challenges that begin with the immediate effects of microgravity. Exposures can trigger rapid physiological changes – such as shifts in immune function, alterations in microbiome profiles, increased viral loads, early bone resorption, and muscle atrophy – that serve as early warning markers. These responses indicate critical thresholds where reversible adaptations might evolve into permanent health issues, underscoring the importance of monitoring rapid bodily changes.

Beyond the physical toll, the psychological and operational challenges of interplanetary travel pose equally significant risks. Prolonged microgravity not only disrupts circadian rhythms and compromises stress responses but also precipitates cognitive and sensorimotor deficits that can impair decision‐making and overall performance. Coupled with the stress of isolation and confinement, these factors may lead to increased mental fatigue and reduced operational efficiency.

By evaluating these issues during short‐duration missions, scientists are gaining crucial insights into the onset of such adaptations and devising effective strategies to enhance mental resilience. This dual focus on physical and psychological well‐being will be key in ensuring that future Mars explorers are equipped to handle the rigorous demands of deep‐space travel. Low earth orbit (LEO) serves as an indispensable proving ground. It allows researchers to simulate and study the spaceflight stresses experienced during extended interplanetary missions, but within a more accessible and controlled environment. Short‐duration missions in LEO enable scientists to test and refine countermeasures such as advanced exercise regimens, artificial gravity protocols, and nutritional or pharmacological interventions. These experiments provide early indicators – like shifts in biomarkers or immediate physiological responses – that inform long‐term mission planning and risk mitigation strategies.

Short‐duration missions to LEO destinations (whether ISS or commercial LEO destinations (CLDs)) offer a unique opportunity to observe rapid physiological and psychological adaptations to microgravity and identify the threshold durations required for significant, potentially permanent changes to occur. A non‐exhaustive list of study topics is available in Table 1. By exploring these, or similar, research questions, short‐duration space missions can provide valuable insights to inform strategies for long‐term mission planning, astronaut training, spacecraft and habitat design, and development of effective countermeasures to maintain astronaut health and performance during future exploration missions.

**TABLE 1:** Potential research areas and questions for commercial LEO missions.

Research area	Examples of research questions
Microbiome and immune system	What short‐term interactions occur between microbiome shifts and immune cell activity during initial exposure to microgravity? How does short‐duration microgravity impact viral loads specifically on the skin and oral cavity within the first few days of flight?
Skeletal and musculoskeletal changes	How early after microgravity exposure does bone resorption begin significantly exceeding formation? Are there rapid, detectable biomarkers for early‐onset bone fragility or muscle atrophy occurring within 7–21 days of microgravity exposure?
Circadian rhythms and heart rate variability	Do circadian disturbances observed in longer missions appear within the first week of spaceflight, and how quickly do astronauts adapt to imposed sleep–wake schedules? Can short‐duration missions establish effective countermeasure protocols (e.g., lighting and feeding schedules) for maintaining circadian alignment from the start of exposure?
Sensorimotor and postural stability	Can short‐duration exposure to altered gravity environments be sufficient to trigger vestibular and sensorimotor adaptations measurable post‐flight? Are certain preflight baseline conditions (fitness levels, balance skills) predictive of rapid sensorimotor recovery?
Sensorimotor performance and haptic feedback	What specific sensorimotor deficits emerge in the first few days of spaceflight, and how rapidly does haptic feedback intervention restore these deficits? Within a short‐duration exposure, how does virtual mass or spring stiffness feedback best support fine motor control?
General physiology and countermeasure efficacy	How quickly do microgravity‐induced physiological change in cardiovascular, muscular, immune, and bone systems become detectable? Are current short‐term countermeasures (e.g., brief daily exercise, specific nutrition supplements) sufficient to delay early physiological decline within missions of 7–21 days?
Cardiac–vascular–respiratory coupling	Can countermeasure body gear effectively sustain normal cardiovascular coupling within a short‐duration mission scenario (7–21 days)? Does a brief exposure to microgravity quickly alter the coupling between respiratory and cardiovascular responses compared to baseline Earth conditions?
Exercise as countermeasure	Are intense, individualized exercise regimens effective at completely preserving musculoskeletal integrity during missions of just 1–3 weeks? What immediate physiological responses (muscle, bone, cardiovascular) to exercise interventions during short‐duration missions are predictive of long‐term countermeasure success?
Physiological and cellular ageing mechanisms	How quickly after microgravity exposure can markers resembling ageing or stress‐induced cellular responses be detected? Are cellular senescence or related stress pathways activated even within very brief microgravity exposure windows?
Sex and gender differences in space adaptation	Do men and women exhibit measurable differences in immune responses, orthostatic tolerance and visual impairments within short‐duration missions? How rapidly do sex‐ or gender‐related physiological differences emerge upon initial exposure to microgravity conditions?
Central nervous system adaptation	Within 7–21 days, do astronauts show measurable changes in cognitive function, motor control, or perception due to central nervous system adaptation? How soon after entering microgravity can neuroimaging techniques detect central nervous system morphological or metabolic adaptations or maladaptations?
Sleep, stress and behavioural health	How rapidly do stress levels, cognitive performance and mood vary within the first week of isolated, confined and microgravity environments? Are shorter‐duration space missions adequate for evaluating effective psychological interventions or supports for maintaining astronaut mental health and cognitive function?
Artificial gravity and adaptation	Could brief daily artificial gravity exposures (within a short mission) maintain normal cardiovascular and musculoskeletal function effectively? How quickly can artificial gravity counteract or mitigate early physiological deconditioning within missions lasting only 7–21 days?
Blood flow, cerebral and muscle oxygenation	Do immediate shifts in muscle blood content occur within short‐duration space missions, and are these associated with performance deficits or orthostatic intolerance upon return? Is muscle oxygen saturation sufficiently sensitive to detect immediate gravitational impact during early microgravity exposure?
Salivary microbiome stability and individuality	How quickly does individual variability in microbiota respond or stabilize within short‐duration isolated environments? Are short missions sufficient for developing personalized microbiome interventions for maintaining health during extended missions?

The current plan is for the ISS to be deorbited in 2030, with the commercial sector to provide access to research opportunities. Currently, commercial human spaceflight missions offered by Axiom dock at the ISS, with the crew planning to remain in orbit for 15 days. Moving beyond the role of providing flights to the ISS, commercial companies plan to launch and maintain multiple potential destinations for human spaceflight (Starlab, Axiom station, Vast Haven‐1/2, Orbital Reef, not to mention other potential future crewed platforms). While the timelines differ for these CLDs, most are expected to be operational by the early 2030s, hopefully avoiding a gap in the presence of pressurized stations. Accompanying these crewed stations in LEO will be automated free flier platforms that launch into LEO then return to earth after some months. And last, but not currently well‐defined, there is the possibility for stations from other agencies in LEO – Russia, India and others.

The LEO ecosystem post‐ISS looks vibrant, especially should all of these platforms and CLDs become operational, and researchers will have a number of options to choose from for space facilities. The mission profile for each one may look slightly different, with different durations of stay and different levels of space stressors experienced at each. Different durations, levels of confinement (based on the local architecture and design of the station), potentially even differing atmospheric characteristics in each could be possible. For example, in the latest Space X commercial spaceflight missions, crew members were confined to the Dragon capsule, whereas for Axiom missions the crew lived and worked in the relatively more spacious environment of the ISS. In a more extreme case, there is the possibility that some stations may also be spun to produce artificial gravity in their outer modules, giving rise to a gravity gradient radially and opening the doors to innovative experimental designs for space physiology.

Another potential change will be the diversity and types of participants for the experiments. Commercial astronauts can be private citizens, members of national space agencies, European astronauts, or employees of the provider companies. This can be seen as ‘double‐edged’ in a sense: more diverse backgrounds for physiological studies and potentially more reflective of the general population, but at the same time, less standardized and potentially with more confounding variables. Moreover, current institutional astronauts have a vast amount of data and health monitoring carried out over the course of their careers – it remains to be seen if the same breadth and depth of data, important for physiological studies, will be collected on private astronauts.

With the plethora of platforms that may exist, more unique mission profiles not currently used during nominal increments might be possible. For example, an initial crew of four could launch to a LEO station, with two returning within 14 days, and the remaining two returning later after another crew docks. Adaptations to gravity transitions could be monitored throughout, with close monitoring of the crew that has returned on the ground. This could provide insights into the body's adaptation mechanisms and their modulation by a gravity signal. Information like this can be critical for designing a mission where crew visit the Martian surface, where an understanding of their needs and capabilities during the adaptation periods will be crucial for keeping them safe. Preparing for Lunar surface missions and the round trip to Mars will also mean derisking the long‐term effects of the space environment on the crew. In a post‐ISS LEO, with many different available crewed platforms, an increase of mission duration past 1 year (approximately the longest time an astronaut has been in orbit) brings us closer to understanding how the human body would adapt to long‐term spaceflight stress. Making these observations in the relatively safe environment of LEO ensures we can protect the crew while maximising the return on scientific knowledge.

Commercial space companies operate at a pace much different to the standard pace within historical space institutions. The authors’ experience with Axiom‐3 (Box 1; Magkos & Puhl, 2024; Puhl et al., 2024) was one of extremely rapid turnaround, relative to previous missions, and a requirement for adaptations to the experimental design and flight readiness processes. At such a pace, it can be foreseen that the number of test subjects could be greatly increased – leading to quicker turnaround on hypothesis testing and stronger statistical power to the conclusions reached. Furthermore, with an increase in flight opportunities in both access and frequency, researchers may feel more comfortable trying more technically risky pilot experiments or technology demonstrations, then further elaborating on the most successful cases. Allowing risk taking of this sort can really unlock scientific innovation and would follow the path many terrestrial labs have followed when making groundbreaking discoveries. Additionally, more flights with more dynamic schedules could allow for ride‐sharing‐like research to be conducted in missions to CLDs.

Focused testing of hypotheses in LEO can lead to better explanatory theories and understanding, enabling targeted studies on the future deep‐space platforms with much more limited access and resources. Furthermore, because of the ease‐of‐access and potentially quicker flight cadence to LEO, the testing and derisking of technologies for crew health monitoring can be done at an increased pace.

## 3 POTENTIAL CHALLENGES IN THE POST‐ISS LANDSCAPE

The plethora of platforms potentially available in the commercial post‐ISS landscape also comes with significant risks. Different mission profiles, while potentially creative, could yield non‐comparable results across studies. Moreover, the different destinations may have differing facilities available, making it more or less possible to run the same study across commercial destinations. A potential risk is to have a multitude of *n* = 1 studies, rather than a coherent set of studies that yield clear results. Such an outcome would drastically slow, if not put an outright stop to, the yield of knowledge needed to bring humans safely to the surface of Mars and return them home.

As with the increased number of platforms, the increased pace in the commercial LEO world carries with it an increased risk of failing to meet optimal scientific objectives. The rapid turnaround anticipated in the future requires a significant outlay of effort and labour on the part of the involved science team. This challenge is made all the more daunting by the drive to include terrestrial labs and small–medium enterprises (SMEs) without previous flight experience – casting them into a new world of unknowns that need to be navigated alongside the running of their labs, teaching, applying for grants, and so on. Without adequate support, these important newcomers may decide they cannot support a space project and instead turn away from the possibility altogether.

## 4 ADAPTING TO NEW LEO

### 4.1 Experimental design and process modularisation

One pathway to navigate this convoluted process is derisking through modularization. Identifying early on a vastness of unknowns, teams need to compartmentalize experimental aspects in parallel to a core experimental process and aims. This allows for dynamic problem solving, and continuation of the research activity, even if certain methods or technologies end up in no‐go scenarios.

Although different teams might decide to face this emerging research environment with different approaches, a modularized and parallelized approach would allow for more scientific output in this new research environment. Researchers could ensure a minimum ‘core’ experiment that consists of the critical measurements, while at the same setting forth additional data points and measurement modalities akin to experimental building blocks depending on the feasibility, experiment needs and destination.

### 4.2 Protocol and measurement standardization from scientific teams: coordination across destinations

To take full advantage of the increased access to space, while controlling the potential variances in the experiments, one potential avenue will be broader scientific collaboration in the form of standardised and potentially open source protocols. These standard protocols would serve to align research projects both on repeated flights to the same destination, and across different destinations in post‐ISS LEO. Examples of these sorts of scientific consortia exist on Earth (International Brain Laboratory; Abbott et al., 2017), where the focus of the consortium is to establish standard hardware and procedure protocols, sample collection methods and analytical techniques, such that any data collected by a member of the consortium can potentially be used by another member of the consortium. Indeed, aboard the ISS there exists something of this sort now in the NASA Complement of Integrated Protocols for Human Exploration Research (CIPHER) study, which aims to establish a holistic picture of the effects of spaceflight on humans by using standard measures and coordination across multiple scientific teams. In the post‐ISS era, with more potential flights and platforms, there will exist the possibility to establish more comprehensive studies of this sort to fully leverage every flight opportunity for useful data. Agencies would have the chance to further bolster all of this data collection by continuing to act as data compliance and archival entities for commercial missions, including data sharing as a requirement for co‐funding.

### 4.3 Hardware and software standardization

Building out conceptually from this idea of standardizing the approach and protocols, the next logical step is to have research modules and modular facilities with standardized hardware, software and interfaces. For research labs on earth, this is so common it has likely gone completely unnoticed as something of critical importance, much as it has done in our personal lives. When I want to write an email, the computer I use does not have to be redesigned from scratch each time and the software written again from scratch. Essentially all of the non‐recurring engineering (design and production that happens on a one‐off basis) has been eliminated from the products we use at home and in the labs. Physical standardization exists in a virtuous circle with protocol standardization – the two reinforce one another, speeding up delivery of results, strengthening comparison across studies, and minimizing risk at the level of fundamental hardware failure. The added benefit for research in space is that a ‘catalogue’ of these standard hardware items could be made available to the wider research community, already qualified for use on orbit, and reducing the time‐to‐flight to take advantage of the increased flight cadence and compressed development timelines. Moreover, along with making the comparison between scientific studies possible, standardized hardware enables interoperability across different experimental instruments – think of how Eppendorf tubes and their dimensions act as a standard sizing for centrifuges, PCR machines, sequencers, and so on.

## 5 CONCLUSIONS

The advent of commercial human spaceflight, combined with the potential availability of multiple different destinations in the near future, opens up new avenues for space life science research. For more than two decades space research has been dominated by a single platform, the ISS, serviced by regular, though infrequent, flights. Access to space has typically proceeded through large national space agencies. With all of this set to change, to open up and provide more routes to researchers in the space environment, many of the challenges for scientific research in space are set to become a thing of the past. Replacing those challenges is an overarching need for coordination, both within the scientific community and at the institutional level when looking to answer specific questions to prepare for a mission to Mars. The challenge is, as always, how to enable innovation, and how to channel the abundance of energy in the directions we seek.

The changing context requires evolution on the part of the different actors: the scientific community, commercial providers and national agencies. International coordination between scientific groups could be mediated by new collaborative groups, such as an International Space Life Sciences Institute or international working groups, who advise careful and precise standardised measurements and protocols of studies. Commercial providers and industry could standardise their hardware and interfaces in collaboration with their customers, albeit possibly mediated by national agencies to ensure no loss of competitive edge. Enabling this transition and evolution could be the national agencies, who have already identified their need to prepare for the upcoming diversification of platforms and more complex environment. Being proactive, agencies can delimit and guide private enterprise by enriching and communicating the requirements for human‐rated commercial missions. Agencies can share their expertise and help to ensure interoperability across platforms in a much more compliance regulator‐type role (similar to the Federal Aviation Administration), while simultaneously driving additional research priorities for deep space exploration.

After a long transit to the ISS, the Axiom‐3 mission was executed flawlessly, returning 100% of the science objectives, and being extended slightly due to poor weather for the return. This mission served to demonstrate that commercial human spaceflight was more than simply space tourism, and meaningful scientific research and technology could be readied for flight quickly. The mission itself was a demonstrator that commercial companies, scientists and space agencies could adapt to quicker timelines together. With the changes that are coming, space is nearer than ever, for everyone.

## AUTHOR CONTRIBUTIONS

Christopher Puhl and Michail Magkos contributed equally to the drafting, writing and editing of this document. Christopher Puhl and Michail Magkos approve the manuscript, are accountable for the integrity of the work, qualify for authorship of the work and are the only authors. The opinions stated here are those of the authors and not necessarily those of their affiliated institutions.

## CONFLICT OF INTEREST

None declared.

### FUNDING INFORMATION

None.
